# A Parasitoid of Aphids Manipulates Host Mummification Site, With Effects on Survival but Not Hyperparasitism

**DOI:** 10.1002/ece3.72764

**Published:** 2026-02-11

**Authors:** Nina Reinmann, Christoph Vorburger, Nina Hafer‐Hahmann

**Affiliations:** ^1^ Institute of Integrative Biology ETH Zürich Zürich Switzerland; ^2^ Eawag Swiss Federal Institute of Aquatic Science and Technology Dübendorf Switzerland; ^3^ Department of Biology University of Konstanz Konstanz Germany

**Keywords:** aphids, body guard manipulation, host behavior, host manipulation, parasitoid wasps

## Abstract

Many parasites change the behavior of their host. Parasitoid wasps, for example, frequently engage in body guard manipulation to induce behaviors in their hosts that enhance parasite survival after pupation. Parasitoids of aphids have repeatedly been found to alter host location on the plant, thereby influencing the location where their host mummifies, that is their pupation site. Potential benefits of this behavior for the parasite, however, remain under debate. Combining experiments in the laboratory and the field, we tested whether the parasitoid *Aphelinus chaonia* induces behavioral changes in its aphid host to influence its mummification location and whether these reduce hyperparasitism, an important source of mortality to the developing parasitoid. *Aphelinus chaonia* clearly caused aphids to move either off the plant or into the leaf axil prior to mummification and host death. However, movement to the leaf axil did not result in any reduction of hyperparasitism. Nevertheless, in the field, mummies situated on the stem were less likely to survive than those elsewhere on the plant, including in the leaf axil. We discuss our findings in the light of potential host manipulation.

## Graphical abstract

Parasitoid wasps can manipulate host behavior to enhance their own survival. Here we find that a parasitoid of aphids, *Aphelinus chaonia*, induces aphids to move to the leaf axil or leave the plant prior to pupation. This seems to have no effect on hyperparasitism, but mummy location may nevertheless impact parasitoid survival.

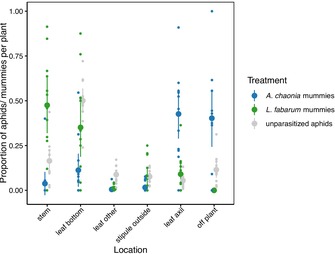

## 1 Introduction

Many parasites change the behavior of their hosts. Most strikingly, parasites use host manipulation to make their hosts behave in a manner that increases their own fitness beyond benefits gained from normal exploitation. Arguably the best‐known examples of such host manipulation come from complex life cycle parasites that make their hosts behave in a manner that increases their susceptibility to predation and thereby facilitates parasite transmission to the next host (Poulin 2010; Moore 2013; Poulin and Maure 2015). The protozoan parasite *Toxoplasma gondii*, for example, causes its intermediate hosts, mice and rats, to lose their innate fear of cats, its definite host (Flegr 2015). Toxoplasmosis may also occur in humans and has been associated with reckless behavior and neurological disorders (Flegr 2013). Parasitoids of insects often engage in bodyguard manipulation, inducing their host to directly or indirectly protect them from natural enemies such as predators and hyperparasitoids, or from other adversities such as bad weather (Maure et al. 2013; Weinersmith 2019). For example, lady bugs stay with their parasitoid wasp mummy after egression and protect it against predation (Maure et al. 2011). Parasitoids can also induce their host to provide protection through more indirect means. Parasitized bumblebees bury themselves in the ground prior to their death and thereby increase parasite survival through protection against adverse conditions and natural enemies (Müller 1994; Malfi et al. 2014).

Aphids are commonly parasitized by parasitoid wasps. These parasitoids inject their eggs into aphids, the larva develops inside the living aphid and eventually kills the aphid prior to pupation, which takes place within a cocoon inside the aphid's empty exoskeleton. This stage is referred to as a mummy and normally remains attached to the plant. Infection by some parasitoid species causes infected aphids to leave the colony prior to mummification and move to different locations on the plant like the leaf top (Brodeur and McNeil 1992) or to leave the plant entirely (Behrendt 1968; Brodeur and McNeil 1989; Müller et al. 1997). This behavior might offer protection to the metamorphosing parasitoid against natural enemies, especially against hyperparasitoids (Brodeur and McNeil 1989, 1992; but see Müller et al. 1997).

In the field, we observed that mummies of the parasitoid *Aphelinus chaonia* seemed to accumulate in the leaf axils of broad bean plants ( *Vicia faba* ), on which their aphid hosts feed. *Aphelinus chaonia* has also been observed to induce aphids to leave plants prior to mummification (Behrendt 1968). We speculated that accumulation in the leaf axils could be an additional case of host manipulation in this species. To qualify as host manipulation, this behavior has to be induced by the parasitoid and, more crucially, it has to provide a fitness benefit to the parasitoid. We propose that such a fitness benefit may arise through reduced predation or hyperparasitism, because the leaf axil is a narrow crevice partially covered by stipules at the leaves' base, potentially offering some protection to the mummy. Hence, we confirmed experimentally that *Aphelinus chaonia* indeed causes changes in the behavior of host aphids that affect the mummification location on the plants towards increased mummification in leaf axils. This led to an increase in parasitoid survival but had no clear effect on rates of hyperparasitism.

## 2 Material and Methods

### 2.1 Hosts, Parasitoids, and Hyperparasitoids

#### 2.1.1 Aphids

We used the black bean aphid (*Aphis fabae fabae*) as host. Black bean aphids are a major pest in sugar beet and broad beans. We worked with one aphid clone that is derived from a single female collected in Altstetten, Switzerland in 2008 (clone ID: A08‐28H‐). Since an antibiotic treatment in 2011, this clone is cured from any known facultative bacterial endosymbionts. The aphids are reared on broad beans (*Vicia faba*) at 18°C–20°C and a 16/8 h light/dark cycle. During the experiment presented here, the rearing temperature was 19°C.

#### 2.1.2 Parasitoids

We used two different parasitoid species, *Aphelinus chaonia* (Hymenoptera: Chalcidoidea: Aphelinidae), the species for which we previously observed the accumulation of mummies in the leaf axils, and *Lysiphlebus fabarum* (Hymenoptera: Ichneumonidea: Braconidae) as a control for any general host reaction to exploitation by a parasitoid. For *L. fabarum* we used multiple asexual lines which have been collected in different locations throughout Switzerland between 2006 and 2009 and have been reared under laboratory conditions ever since. *Aphelinus chaonia* was collected in different places in Kanton Zürich, Switzerland, from May–June 2020 to establish a laboratory population. In each generation between 100 and 200 individuals of *A. chaonia* were transferred to cages with new plants and new aphids. Both species were maintained in insect rearing cages (32.5 × 32.5 × 32.5 cm; BugDorm‐4F3030; MegaView Science) on black bean aphids and broad bean plants at 19°C and a 16/8 h light/dark cycle. *Aphelinus chaonia* was temporarily reared at 22°C to speed up development. Both species are frequent and important parasitoids of black bean aphids in natural populations (Rothacher et al. 2016; Hafer‐Hahmann and Vorburger 2021; Narayan et al. 2022; Gimmi et al. 2023).

#### 2.1.3 Hyperparasitoids

To study mummy susceptibility to hyperparasitoids in the lab, we used a single species, *Pachyneuron aphidis* (Hymenoptera: Chalcidoidea: Pteromalidae). It frequently occurs in the field on parasitoids of *A. fabae* (Rothacher et al. 2016; Hafer‐Hahmann and Vorburger 2021; Narayan et al. 2022). *Pachyneuron aphidis* is a mummy parasitoid that attacks its hosts at the pupal stage. We collected it in September 2020 by placing plants with aphids parasitized by *L. fabarum* in an outdoor site with natural vegetation on the Eawag campus in Dübendorf, Switzerland. The plants with hyperparasitized aphids were returned to the lab until the hyperparasitoids hatched. From the hatching *P. aphidis* , we founded a laboratory population that was maintained in rearing cages (BugDorm‐4F3030; MegaView Science) on aphids parasitized by *L. fabarum* at 19°C and a 16/8 h light/dark cycle. Hyperparasitoids were transferred to new cages with fresh plants and hosts in each generation. Experiments took place in late 2020 and early 2021 after they had been in the lab for only a few generations.

### 2.2 Experimental Procedures

#### 2.2.1 Mummification Location

We tested whether under laboratory conditions, an infection with *A. chaonia* affected the location where aphids mummified in comparison to where unparasitized aphids or aphids mummified by another parasitoid, *L. fabarum*, were located on the plant. To set up the experiment, two adult aphids were added to each of 36 (12 per treatment) individually potted 7‐day old broad bean plants and removed 3 days later. The next day we counted all nymphs produced by these adults and exposed them to one of three treatments: Addition of 5 *A. chaonia* (haphazard mixture of males and females; treatment of interest), addition of two *L. fabarum* females (asexual lines; control for any general reaction of aphids to parasitoids and mummification), or no addition of wasps (control without parasitoids). Throughout the experiment, aphids and wasps were confined on the plant with a cellophane bag.

**FIGURE 1:**
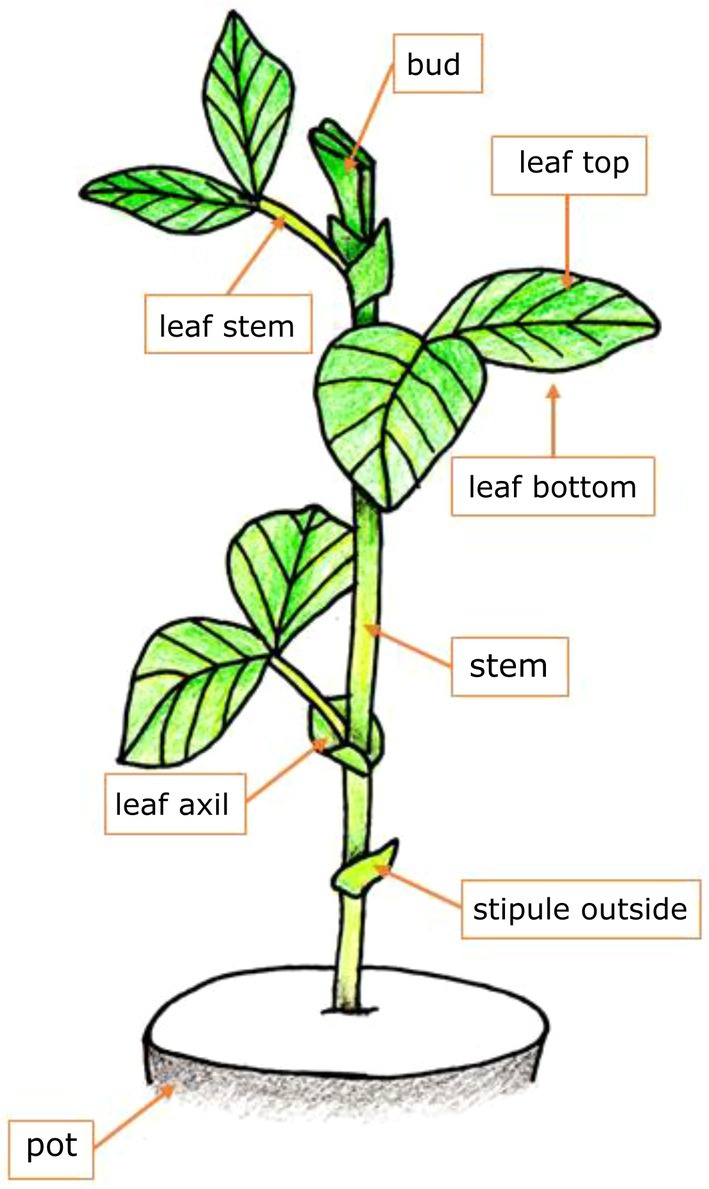
Broad bean plant with possible mummy/aphid locations. In addition to the locations shown in this figure, we recorded the location “bag” for aphids/mummies that had moved to the cellophane bag covering the plant. Drawing of bean plant by Corinne Hertäg, modified by Nina Reinmann.

We defined nine different locations in which aphids or mummies could be situated (Figure 1), namely: bag (off the plant and on the cellophane bag; under natural conditions these individuals would have left the plant), pot (in nature these individuals would have left the plant or mummified on the ground), stipule outside, leaf axil (the location where we had previously observed an increased number of *A. chaonia* mummies), stem, leaf bottom, leaf top, leaf stem, bud. On each plant, we counted the number of mummies and healthy aphids in each of these locations 9 to 10 days after exposure to parasitoid wasps.

#### 2.2.2 Susceptibility to Hyperparasitoids: Lab Experiment

We tested whether mummy location influenced the parasitoid mortality from the hyperparasitoid *P. aphidis* . Due to a limited amount of *P. aphidis* available per generation, the experiment was conducted in two rounds. To obtain mummies for the experiment, we added adult aphids to young broad bean plants (3 aphids per plant) and allowed them to reproduce for 3 days, after which they were removed. The next day we added 5 *A. chaonia* for 2 days to each plant and waited another 11 days (round 1) or 9 days (round 2) for mummies to form. We obtained *L. fabarum* mummies in the same manner using different plants, but only added two wasps and started 3 days later (round 1) in order to obtain mummies from both species on the same day despite the shorter development time of *L. fabarum*. In round 2, we waited 10 days for mummies of *L. fabarum* to form after removal of the parasitoids and mummies of *L. fabarum* and *A. chaonia* were processed on a different day (see below).

To prepare plants with mummies for exposure to hyperparasitoids, we first removed all mummies from the plants and subsequently reattached 12 mummies to four predefined locations on each plant, that is, stem, leaf axil, leaf top, and leaf bottom (3 mummies per location). If we were unable to obtain enough mummies from one plant, we supplemented with mummies from other plants. Reattachment consisted of gluing the mummies to the plants using glue normally used by beekeepers to mark bees (Opalith Königinnen Zeichenleim, APILINE GmbH, Erlenbach, Switzerland), which should not have any adverse effects on insects. Afterwards, the plants were stored for a day in a big insect cage to let the glue dry properly. Due to the loss of some replicates during set up and an insufficient number of mummies and hyperparasitoids, we were only able to set up 17 replicates with *A. chaonia* mummies and 7 with *L. fabarum* mummies. The manipulated plants were individually covered with a cellophane bag and four to five *P. aphidis* (haphazard mixture of males and females in round 1 and 3 females and 1–2 males in round 2) were added. They were left undisturbed for 18–20 h at 19°C, which included an 8 h dark period. Afterwards, the mummies were collected and stored in insect breeding dishes. Mummies of the same plant and location were stored together. About 5 weeks later when all mummies should have hatched, we examined the mummies and the hatched parasitoids and hyperparasitoids under a stereo microscope to determine the fate of each mummy.

#### 2.2.3 Susceptibility to Hyperparasitoids: Field Experiment

To test the effect of mummy location on the risk of hyperparasitism in a natural hyperparasitoid community, we exposed mummies glued to different locations on broad bean plants under field conditions. Aphids were reared as described above on 12 plants and exposed to *A. chaonia*. After mummification, the mummies were removed (11 days after exposure to *A. chaonia*), and their location was manipulated by gluing them back onto the same plants in the four specific locations also used for the lab experiment (stem, leaf axil, leaf top, and leaf bottom). Some plants were supplemented with mummies from other plants or from *A. chaonia* breeding cages when not enough mummies could be obtained from a particular plant. We photographed each plant to document mummy locations. The plants were labeled, and four plants were planted in each of three square plastic plant pots (34 × 34 × 32 cm, with water reservoir). These pots were placed outdoors on the campus of Eawag in Dübendorf, Switzerland, in a fenced area covered by natural vegetation. They were left outside for a week, accessible to the natural community of natural enemies, ants, and other small animals. We only restricted access of snails and slugs as far as possible. Field exposure took place in late September of 2020 (daily mean temperature: 15°C–20°C, no significant rainfall (MeteoSchweiz 2020)). Afterwards, the plants were collected, and the mummies initially examined on the plant under a stereo microscope to assess any losses of mummies, hatching that had already occurred, and any sign of predation. Subsequently, intact mummies were collected and stored in insect dishes (mummies from the same plant and location stored together). These insect dishes were checked regularly for hatched wasps, which were determined as either parasitoids (*A. chaonia*) or hyperparasitoids (identified at least to the genus level) under a stereo microscope.

### 2.3 Statistical Analysis

Statistical analyses were conducted in Rstudio version 2023.6.1.524 (RStudio Team 2020) using R version 4.3.0 (R Core Team 2023), and plots were generated using ggplot2 (Wickham 2016).

Prior to analyzing the locations where aphids mummified, we removed one location and aggregated other locations to avoid problems with complete separation of data among treatments later in our analyses. Specifically, we removed the location “bud,” because only 30 adult aphids and not a single mummy were recorded there, we aggregated counts on “leaf top” and “leaf stem” to “leaf other” and counts on “pot” and “bag” to “off plant” (see Table S1).

To analyze the effect of parasitoid infection on where aphids mummified, we first compared the locations of live aphids in the no parasitoid treatment with the locations of mummies in the two parasitoid treatments, ignoring any aphids that remained unparasitized in the two parasitoid treatments. For this we compared the numbers of aphids or mummies at the six locations on each plant (i.e., our response variable was a vector with the numbers of individuals at each location, with one row in the dataset per plant and plants representing the independent replicates), using multinomial log‐linear models from the R package VGAM (i.e., vector generalized linear model with family multinomial; Yee 2025) followed by Anova from the package car (Fox and Weisberg 2019) to obtain *p*‐values. Since we observed a significant effect of treatment, we built additional models for each pairwise comparison (*A. chaonia* versus no parasitoid, *A. chaonia* versus *L. fabarum*, and *L. fabarum* versus no parasitoid) as a post hoc test to identify between which treatments these significant differences occurred. We calculated Bonferroni‐adjusted *p*‐values to account for multiple testing. Additionally, we analyzed each location on the plants separately by applying generalized linear mixed models (GLMM) with binomial errors (glmer from package lme4, (Bates et al. 2015)) to the following vector: (n aphids/mummies at this location, n aphids/mummies at all other locations on the plant). A replicate‐level random effect (i.e., plant) was included to account for overdispersion of our data. Because no mummies of *L. fabarum* were found off the plant, we added a “fake” single mummy at location “off plant” to 5 replicates of the *L. fabarum* treatment in the dataset to avoid problems with model convergence. This data manipulation reduced the true differences among treatments and therefore allowed us to obtain a conservative *p*‐value. For all locations showing a significant treatment effect after Bonferroni correction we subsequently conducted Tukey post hoc tests for pairwise comparisons (glht, package multcomp (Hothorn et al. 2008)).

The fact that not all aphids were parasitized in the two wasp treatments provided an additional opportunity to test for parasitoid manipulation of host location. For the *A. chaonia* and the *L. fabarum* treatments we therefore also compared the numbers of mummies and unparasitized aphids at the six locations on the same plants, again using multinomial models.

To assess the influence of mummification location on the rate of hyperparasitism by *P. aphidis* in the laboratory experiment, we only considered mummies from which a wasp hatched. Our response was the probability of parasitoid survival, that is, the probability that the emerging wasp was a parasitoid rather than a hyperparasitoid (success (parasitoid hatched), failure (hyperparasitoid hatched)), that is, one data point with data on success/failure per plant and location. This was analyzed using a GLMM with binomial errors, testing for the effects of location on the plant, parasitoid species, the location‐by‐species interaction, as well as the experimental round (the experiment was carried out in two test rounds). Plant identity was included as a random effect.

In contrast to the laboratory experiment, not all mummies glued to the plants could be retrieved after outdoors exposure in the field experiment. A first analysis considered only the retrieved mummies from which a wasp had emerged, again using a binomial GLMM to test for the effect of mummy location on the probability that a parasitoid (*A. chaonia*) rather than a hyperparasitoid emerged, including plant identity nested within pot identity as random effects. In addition, we ran two binomial GLMMs considering all mummies that we glued to the plant, one testing if its location affected whether a mummy could be recovered after exposure in the field, and a second model testing whether mummy location affected the survival of the parasitoid, where nonsurvival represented all other fates, that is, loss of the mummy, the hatching of a hyperparasitoid, or failure to hatch. Again, Anova from the package car (Fox and Weisberg 2019) was used to obtain *p*‐values, and significant effects of mummy location were followed up with Tukey post hoc tests for pairwise comparisons (glht, package multcomp, Hothorn et al. 2008).

## 3 Results

### 3.1 Does *A. chaonia* Change Mummification Location?

**FIGURE 2:**
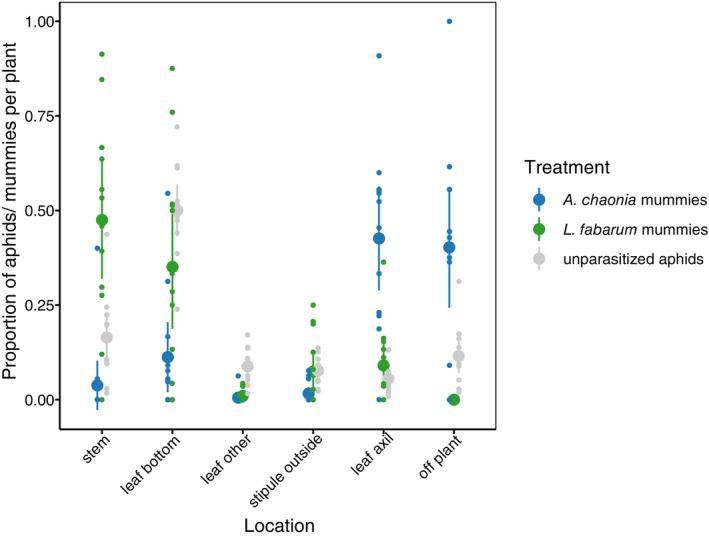
Proportion of aphids or mummies found at different locations on the plant. Large dots represent means and error bars represent 95% CI; small dots represent individual plants. *N* = 36 plants (12 per treatment).

Parasitoid treatment had a significant influence on where aphids mummified and died (Multinomial log‐linear model; df = 10; *χ* ^2^ = 3553; *p* < 0.0001, Figure 2, Table S2). This was the case also for any of our pairwise comparisons (*A. chaonia* versus *L. fabarum*: df = 5; *χ* ^2^ = 254; *p* < 0.0001; *A. chaonia* versus no parasitoid: df = 5; *χ* ^2^ = 104; *p* < 0.0001; *L. fabarum* versus no parasitoid: df = 5; *χ* ^2^ = 100; *p* < 0.0001, Table S2). Comparing the location of mummies and surviving aphids within the two treatments that were exposed to parasitoids showed a similar pattern. The locations of mummies and surviving aphids differed significantly in either parasitoid treatment (*A. chaonia*: df = 5, *χ* ^2^ = 207. *p* < 0.0001, Figure S1A, Table S3A; *L. fabarum*: df = 5, *χ* ^2^ = 24, *p* = 0.0002, Figure S1B, Table S3B), albeit these seem more pronounced in *A. chaonia*.

What caused the overall differences became clear when we analyzed each location separately. After adjusting for multiple testing, we found significant differences for all locations except the stipule outside (Table 1, Table S4). *Aphelinus chaonia* infected aphids moved off the plant prior to mummification or mummified in the leaf axil more frequently than both unparasitized aphids and *L. fabarum* infected aphids (Figure 2, Table 1). Also, unparasitized aphids moved off the plant more frequently than *L. fabarum* infected aphids, which did not show this behavior (Figure 2). However, the propensity to leave the plant was less pronounced than in *A. chaonia* infected aphids, and they were found in different locations: all unparasitized aphids found off the plant had moved to the bag, whereas two thirds of the *A. chaonia* mummies were found on the pot (Table S1). By contrast, the number of *A. chaonia* mummies found on the bottom of leaves and the stem—the locations preferred by unparasitized aphids and *L. fabarum* mummies—was strongly reduced compared to the other two treatments (Figure 2, Table 1). Our analyses of individual locations also revealed additional differences between *L. fabarum* mummies compared to unparasitized aphids; *L. fabarum* mummies occurred more frequently on the plant stem and less frequently on “leaf other” (Figure 2, Table 1).

**TABLE 1:** Outcome of generalized linear mixed models to analyze the effect of treatment on the location of aphids/mummies at each of six locations on the plants.

Location	*χ* ^2^ (df = 2)	*p* (adjusted *p*)	Post hoc comparisons
Stem	34.67	< 0.001 (< 0.001)	**Lfab‐Acha: Estimate = 4.32 ± 0.79; *z* = 5.51; *p* < 0.001** **NoPa‐Acha: Estimate = 2.55 ± 0.77; *z* = 3.31; *p* = 0.003** **NoPa‐Lfab: Estimate = −1.77 ± 0.47; *z* = −3.78; *p* < 0.001**
Leaf bottom	25.98	< 0.001 (< 0.001)	**Lfab‐Acha: Estimate = 1.62 ± 0.49; *z* = 3.34; *p* = 0.002** **NoPa‐Acha: Estimate = 2.38 ± 0.47; *z* = 5.10; *p* < 0.001** NoPa‐Lfab: Estimate = 0.76 ± 0.40; *z* = 1.91; *p* = 0.136
Leaf other	18.00	< 0.001 (0.001)	Lfab‐Acha: Estimate = 0.70 ± 0.40; *z* = 1.91; *p* = 0.81 **NoPa‐Acha: Estimate = 2.83 ± 1.03; *z* = 2.74; *p* = 0.016** **NoPa‐Lfab: Estimate = 2.13 ± 0.63; *z* = 3.36; *p* = 0.002**
Stipule outside	6.04	0.049 (0.293)	
Leaf axil	55.81	< 0.001 (< 0.001)	**Lfab‐Acha: Estimate = −2.30 ± 0.45; *z* = −5.11; *p* > 0.001** **NoPa‐Acha: Estimate = −2.72±; *z* = −7.25; *p* < 0.001** NoPa‐Lfab: Estimate = −0.42 ± 0.42; *z* = −0.99; *p* = 0.583
Off plant	46.86	< 0.001 (< 0.001)	**Lfab‐Acha: Estimate = −3.74 ± 0.60; *z* = −6.25; *p* < 0.001** **NoPa‐Acha: Estimate = −1.84 ± 0.38; *z* = −4.92; *p* < 0.001** **NoPa‐Lfab: Estimate = 1.89 ± 0.58; *z* = 3.28; *p* = 0.003**

*Note:* Please refer to Table S4 for further details on these models. Estimate represents estimate ±SE. Lfab, *L. fabarum*; Acha, *A. chaonia*; noPa, no parasitoid. Significant pairwise comparisons in posthoc tests have been highlighted in bold. *N* = 36 plants (12 per treatment).

### 3.2 Do Different Mummy Locations Alter Susceptibility to Hyperparasitoids?

We found no clear indication that the location of mummies had any effect on hyperparasitism, neither when exposed to a single hyperparasitoid species under laboratory conditions nor when exposed to a natural hyperparasitoid community in the field.

**FIGURE 3:**
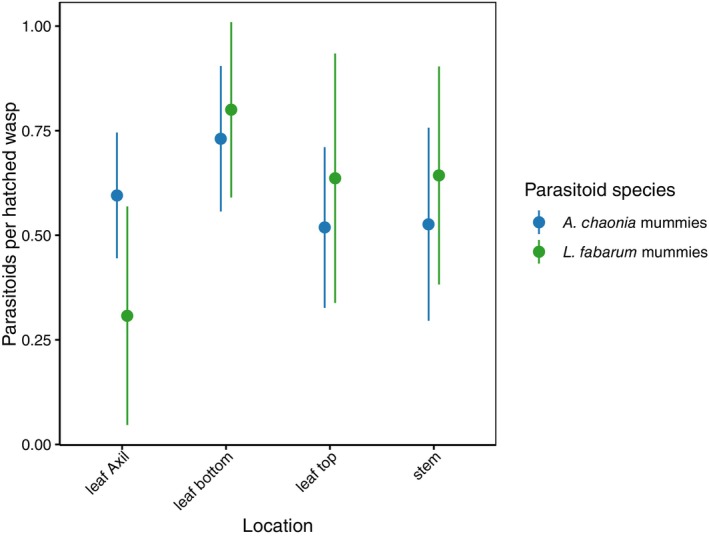
Rates of parasitoid survival after exposure to *P. aphidis* based on mummy location in the lab. Sample sizes at each location given as *A. chaonia* mummies (plants with *A. chaonia* mummies)/*L. fabarum* mummies (plants with *L. fabarum* mummies): Leaf Axil: 42 (17)/13 (7), Leaf bottom: 26 (13)/15 (7), Leaf top: 27 (15)/11 (5), Stem: 19 (10)/14 (7). Error bars represent 95% CI.

In the laboratory, somewhat less than half of the hatching wasps on average were hyperparasitoids ( *P. aphidis* ). While there was a significant difference between the two rounds of the experiment we executed (binomial GLMM: df = 1, *χ* ^2^ = 16.71, *p* < 0.0001), there was no significant variation in parasitoid survival among the four locations at which mummies were presented (df = 3, *χ* ^2^ = 3.81, *p* = 0.2832), no difference between parasitoid species (*A. chaonia* vs. *L. fabarum*, df = 1, *χ* ^2^ = 0.61, *p* = 0.4322), nor was there a significant location‐by‐species interaction (df = 3, *χ* ^2^ = 6.01, *p* = 0.1112) (Table S5, Figure 3).

**FIGURE 4:**
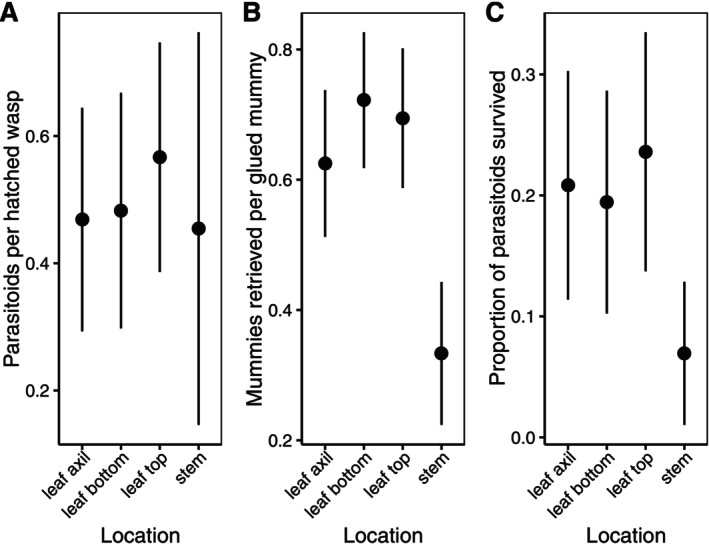
Fate of mummies in the field by mummy location. (A) Proportion of parasitoids hatched out of all hatched wasps; (B) Proportion of mummies retrieved out of all mummies glued to plants; (C) Proportion of parasitoids that survived to hatch out of all mummies glued. Sample sizes for figure A given as mummies hatched (plants on which mummies hatched) at each location: Leaf Axil: 33 (10), Leaf bottom: 29 (12), Leaf top: 30 (11), Stem: 11 (8). For figure B–C, always 72 mummies per location were glued, distributed equally over 12 plants. Error bars represent 95% CI.

In the field experiment, hyperparasitoids hatched from approximately half of the *A. chaonia* mummies we could retrieve. With the exception of a single individual each of *Dendrocerus* sp. and *Alloxysta* sp., all hyperparasitoids were either *P. aphidis* (*n* = 26)—the species used in the lab—or *Syrphophagus aphidivorus* (*n* = 24). Just as in the laboratory experiment, there was no significant variation in parasitoid survival per hatched wasp among the four locations at which mummies were attached on the plants (df = 3, *χ* ^2^ = 1.62, *p* = 0.6539, Table S6A, Figure 4A).

We were only able to recover part of the mummies that we glued onto the plants and put in the field. The others were lost, possibly to predation or removed by physical influences (wind, weather). Hence, we also analyzed the proportion of mummies we were able to retrieve, and this was clearly influenced by mummy location (df = 3, *χ* ^2^ = 27.43, *p* < 0.0001, Table S6B, Figure 4B); mummies glued to the stem were less frequently retrieved than mummies glued to any other location (*p* < 0.003; Table 2). When we looked at overall parasitoid survival, that is the proportion of mummies from which a parasitoid hatched out of all mummies glued, we saw a similar, albeit weaker pattern (Figure 4C), in that mummy location significantly influenced the survival of *A. chaonia* (df = 3, *χ* ^2^ = 7.89, *p* = 0.0484, Table S6C, Figure 4C). Again, survival was reduced for parasitoids from mummies glued to the stem (Figure 4C) and this was significant in the pairwise comparison with leaf top (*p* = 0.031) and marginally significant in the comparison with leaf axil (*p* = 0.077; see Table 2).

**TABLE 2:** Pairwise comparisons for the effect of mummification location on mummy retrieval (A) and overall parasitoid survival (B).

	A: Mummy retrieval			B: Parasitoid survival		
	Estimate ± SE	*z*	*p*	Estimate ± SE	*z*	*p*
Leaf bottom‐ leaf axil	0.47 ± 0.37	1.29	0.575	−0.09 ± 0.43	−0.22	0.9963
Leaf top—leaf axil	0.33 ± 0.36	0.91	0.801	0.17 ± 0.42	0.42	0.9745
Stem‐ leaf axil	−1.29 ± 0.36	−3.55	**0.002**	−1.3 ± 0.55	−2.39	*0.0769*
Leaf top – leaf bottom	−0.14 ± 0.38	−0.38	0.981	0.27 ± 0.42	0.64	0.9187
Stem – leaf bottom	−1.76 ± 0.38	−4.65	**< 0.001**	−1.23 ± 0.56	−2.21	0.1182
Stem – leaf top	−1.62 ± 0.37	−4.34	**< 0.001**	−1.50 ± 0.55	−2.74	**0.0310**

*Note:* Significant *p*‐values have been highlighted in bold; marginally non‐significant ones in italic.

## 4 Discussion

We find that most aphids infected by the parasitoid wasp *Aphelinus chaonia* get mummified either off the plant or within the plant's leaf axil. These locations are markedly different from those of unparasitized aphids and those of aphids mummified by a different parasitoid species, *L. fabarum*. This implies that, directly or indirectly, *A. chaonia* causes its aphid host to seek out the leaf axil or leave the plant prior to mummification. Plant leaving behavior has also been observed in other aphids parasitized by different parasitoid species (Behrendt 1968; Brodeur and McNeil 1989, 1990, 1992; Chow and Mackauer 1999; Singh and Marjorie 2007; Khudr et al. 2013). At least in some species, including *L. fabarum* (Behrendt 1968), this behavior seems to be associated exclusively with diapause or aestivation of the parasitoid (Behrendt 1968; Brodeur and McNeil 1989, 1990, 1992). Other parasitoids cause their aphid host to leave their colony and move elsewhere on the plant (Brodeur and McNeil 1992; Müller et al. 1997; Lagos et al. 2001). Whether these behaviors are adaptive for the parasite in the sense of host manipulation remains under debate.

For the case of *A. chaonia* reported here, we cannot provide a conclusive answer to this question, either. We found no evidence that mummifying at a seemingly protected site, the leaf axil, reduces hyperparasitism. On the other hand, mummifying on the stem seems to entail an increased loss of mummies under field conditions. The plant stem is a location avoided actively by *A. chaonia*‐parasitized aphids because they occur there less frequently than unparasitized aphids (Table 1). We do not know precisely why we retrieved so few mummies from the stem in the field experiment. Avoiding predation has been suggested as a possible benefit of host manipulation by parasitoids in aphids (Brodeur and McNeil 1992; Müller et al. 1997), but—to our knowledge—clear evidence is still missing. We did not explicitly address the effect of predation in our study. However, the plants with mummies exposed in the field were freely accessible to predators. While we did not observe any clear signs of predation in the present experiment, we observed high rates of predation on aphids and mummies in a previous experiment that also employed potted plants at the same field site, sometimes resulting in a complete loss of colonies (Narayan et al. 2022). So, predation may well have contributed to the loss of mummies in the field. Since we glued mummies to the four locations on the plants compared in the field experiment, we cannot entirely rule out handling artifacts, for example, not attaching them properly to the plant stem. However, since we lost no mummies that were glued onto the stem in the exact same manner for the lab experiment, it is unlikely to be the only reason. It is possible that mummies on the stem might be most exposed to the elements such as rain and wind, even though the weather was mostly fine during our experiment (MeteoSchweiz 2020). Finding sites that protect them from the elements and adverse weather has indeed been proposed as a possible benefit parasitoids may gain by mummifying in more protected locations (Brodeur and McNeil 1989, 1990; Müller et al. 1997), albeit, to our knowledge, never explicitly tested. Overall, even taking these losses into account, we find no advantage for *A. chaonia* of mummifying in the leaf axil versus any other location on the plant, with the possible exception of the stem (Table 2).

We also observed differences between the location of *L. fabarum* mummies and unparasitized aphids, especially an increased number of *L. fabarum* on the stem and an increased number of unparasitized aphids off the plants (Figure 3). The latter was likely due to the higher numbers of aphids on plants not exposed to parasitoids, causing aphids to leave plants in search of new food sources.

A behavioral change that takes place upon infection or during the development of a parasite need not be host manipulation that benefits the parasite. Infections can also result in side effects that alter host behavior. For example, parasitoid exploitation could cause massive disruption to the host, accidentally resulting in altered behavior. Differences between different parasitoids could then result from variation in how parasitoids exploit their host (Müller et al. 1997; Chow and Mackauer 1999). It would be surprising, however, that some parasitoids (such as *A. chaonia*) induce such strong behavioral side effects while others—such as *L. fabarum*—do not or only when diapausing. Such variation makes an adaptive explanation more likely, albeit not necessarily for the parasite. Behavioral changes upon an infection could also represent host adaptations to cope with the infection and limit its fitness loss. Such a strategy seems implausible if we consider an individual aphid; once a parasitized aphid is close to mummification, its residual individual fitness is zero. It can no longer reproduce, and it will get killed by the parasitoid. However, aphid colonies usually consist of very few or even a single clone (Vantaux et al. 2011). Hence, if by altering its behavior an aphid could increase the fitness of other members of its colony, for example, by reducing parasitoid survival or moving away from the colony to reduce the risk that the emerging parasitoid will attack clone mates, such a behavior could be adaptive via inclusive fitness benefits. It has been proposed that even suicide of infected animals could be adaptive in a system with very high relatedness, such as clonal aphids, through the empirical evidence is questionable at best (reviewed by Humphreys and Ruxton 2019). Aphid colonies are often very short lived (Winder et al. 2014). Hence, by the time the parasitoid hatches and is ready to attack new aphids, the colony may no longer exist at the same location or a new colony (of potentially unrelated individuals) may have formed. This is especially true for parasitoids with long developmental times, such as *A. chaonia*, or when parasitoids undergo diapause.

Understanding the underlying mechanism may help us distinguish whether a trait of infected animals evolved as a host manipulation to benefit the parasite, as a mere side effect, or an adaptive response of the host. In the parasitoid *Aphidius ervi*, parasitoid genotype significantly influences where aphids mummify (Khudr et al. 2013). This strongly suggests that the parasitoid has at least some influence on the behavior of the parasitized aphid. A closer look at some other systems may allow us to speculate about potential underlying mechanisms. The parasitoid wasp *Dinocampus coccinellae* parasitizes lady bugs, but rather than killing them, it induces them to stay with the parasitoid even after eggression and pupation to defend the parasitoid pupae. Prior to eggression, it transmits a virus to its host. The presence of this virus is crucial for whether or not host manipulation occurs, indicating it may be the virus rather than the parasitoid that causes the altered host behavior (Dheilly et al. 2015). More recently, a related virus has been identified in *L. fabarum* (Lüthi et al. 2020). Whether this virus and similar viruses in other aphid parasitoids might be involved in host manipulation, or how it could do so, has not been studied to date. A better understanding of these mechanisms could also help us explain the differences we observed in host manipulation between *L. fabarum* and *A. chaonia*. Parasites often manipulate their host by exploiting pre‐existing pathways. For example, a virus of caterpillars can affect the molting pathway of its host and cause it to move up in the vegetation to die, arguably to facilitate parasite spread to new hosts (Hoover et al. 2011). In some aphid species, molting site corresponds to mummification side (Müller et al. 1997). This raises the possibility that pathways involved in molting could be potential targets to manipulation also in aphids—or suggest that parasitoid exploitation accidentally induces molting behavior, as previously speculated (Müller et al. 1997). Either way, if such a behavioral modification results in a fitness benefit for the parasitoid, selection would act on it and favor its spread in parasitoids, irrespective of its original purpose.

Parasites that share similar life cycles and transmission strategies have often developed strikingly similar host manipulation independently—for example, fungi and viruses induce summiting disease to facilitate the spread of infective particles (reviewed by Masoudi et al. 2024). At the same time, different populations of the same parasite species can induce different levels of host manipulation in the same host species (Franceschi et al. 2010; Hafer 2018). Here we studied two parasitoids that share the same host and would both benefit from high pupal survival, yet they differ in their behavioral effect on their aphid host. Such differences have previously been noted. Parasitoids that develop more slowly may stand to gain more from inducing their host to seek out protective sites since the time they spend as a highly vulnerable pupa is extended (Chow and Mackauer 1999). While our findings—manipulation in the slower developing *A. chaonia*, none in the faster developing *L. fabarum*—would be in line with this hypothesis, this pattern seems not to hold for all species (Chow and Mackauer 1999). Nevertheless, ecology seems a likely determinant of host manipulation in parasitoids, albeit the ecological factors driving it require further study. At the same time, host manipulation has ecological consequences that can go well beyond the species involved. The most striking examples of this come from different helminths that strengthen or even create trophic links causing bottom‐up and top‐down effects in food webs (reviewed by Sato et al. 2019). Potential ecological effects of parasitoids that protect their host from natural enemies remain largely unexplored.

## Author Contributions

**Nina Reinmann:** conceptualization (supporting), formal analysis (supporting), investigation (lead), writing – original draft (equal). **Christoph Vorburger:** conceptualization (supporting), investigation (supporting), writing – review and editing (equal). **Nina Hafer‐Hahmann:** conceptualization (lead), formal analysis (lead), investigation (supporting), writing – original draft (equal), writing – review and editing (equal).

## Funding

This work was supported by Schweizerischer Nationalfonds zur Förderung der Wissenschaftlichen Forschung (21003A_181969, CRSII3_154396).

## Conflicts of Interest

The authors declare no conflicts of interest.

## Supporting information


**Table S1:** ece372764‐sup‐0001‐TablesS1‐S6.docx.

**Figure S1:** ece372764‐sup‐0002‐FigureS1.pdf.

## Data Availability

Data and code are available through zenodo (https://doi.org/10.5281/zenodo.15854009).
