# Supplementary material for: A Parasitoid of Aphids Manipulates Host Mummification Site, With Effects on Survival but Not Hyperparasitism
Source: Ecol Evol. 2026 Feb 11;16(2):e72764. doi: 10.1002/ece3.72764 (PMC12894770; doi:10.1002/ece3.72764)
Supplement: Supplementary file 1 — Table S1: ece372764‐sup‐0001‐TablesS1‐S6.docx. [file ECE3-16-e72764-s001.docx]

# Appendices

**Table S1: Number of aphids and mummies in each position.** Position “leaf other” comprises of mummies and aphid located on leaf stem and leaf top (relatively few aphids and especially mummies in either position), position “off plant comprised of position “pot” and “bag” (“pot” was only existent in the A. chaonia treatment). Position “bud” was removed (no mummies in any treatment and few aphids). Data based on 36 plants (12 per treatment).

|  | ***Aphelinus chaonia*** | | ***Lysiphlebus fabarum*** | | **No parasitoid** |
| --- | --- | --- | --- | --- | --- |
| **Position** | **N mummies (mean +/- se)** | **N aphids (mean +/- se)** | **N mummies (mean +/- se)** | **N aphids (mean +/- se)** | **N aphids (mean +/- se)** |
| Leaf bottom | 0.73+/-0.31 | 8.88+/-2.38 | 3.52+/-1.01 | 8.85+/-2.35 | 49.34+/-7.21 |
| Leaf other | 0.04+/-0.04 | 2.92+/-0.56 | 0.17+/-0.09 | 1.83+/-0.35 | 5.67+/-0.70 |
| Off plant | 3.84+/-0.67 | 0.89+/-0.74 | 0 | 4.33+/-3.33 | 34.42+/-5.64 |
| Stem | 0.12+/-0.09 | 8.17+/-1.95 | 3.44+/-0.49 | 8.76+/-2.44 | 13.82+/-2.54 |
| Stipule inside | 1.89+/-0.28 | 2.37+/-0.67 | 0.74+/-0.2 | 3.48+/-0.71 | 4.82+/-0.63 |
| Stipule outside | 0.13+/-0.07 | 5.83+/-1.38 | 0.71+/-0.24 | 4.86+/-0.94 | 5.53+/-0.70 |

**Table S2: Summary of multinom model for aphid/ mummy positions and for each pairwise comparison.** Values are coefficients +/- se. Comparison to position stipule inside and *A. chaonia* (overall only).

**Overall:**

| **Level** | **Estimate** | **SE** | **z** | **p** |
| --- | --- | --- | --- | --- |
| (Intercept): leaf bottom | -1.33 | 0.26 | -5.17 | <0.01 |
| (Intercept): leaf other | -4.28 | 1.01 | -4.25 | <0.01 |
| (Intercept): off plant | 0.01 | 0.17 | 0.08 | 0.93 |
| (Intercept): stem | -3.18 | 0.59 | -5.39 | <0.01 |
| (Intercept): stipule outside | -3.18 | 0.59 | -5.39 | <0.01 |
| Lysiphlebus: leaf bottom | 2.89 | 0.36 | 8.11 | <0.01 |
| Lysiphlebus: leaf other | 2.38 | 1.18 | 2.01 | 0.04 |
| Lysiphlebus: off plant | -15.56 | 322.37 | -0.05 | 0.96 |
| Lysiphlebus: stem | 4.94 | 0.64 | 7.76 | <0.01 |
| Lysiphlebus: stipule outside | 3.18 | 0.67 | 4.75 | <0.01 |
| No parasitoid: leaf bottom | 3.63 | 0.27 | 13.50 | <0.01 |
| No parasitoid: leaf other | 4.89 | 1.01 | 4.83 | <0.01 |
| No parasitoid: off plant | 0.77 | 0.19 | 4.11 | <0.01 |
| No parasitoid: stem | 4.37 | 0.60 | 7.35 | <0.01 |
| No parasitoid: stipule outside | 3.54 | 0.60 | 5.94 | <0.01 |

**Pairwise:**

| **Comparison** | **Level** | **Estimate** | **SE** | **z** | **p** |
| --- | --- | --- | --- | --- | --- |
| Aph-No | (Intercept): leaf bottom | -1.33 | 0.26 | -5.17 | <0.01 |
| Aph-No | (Intercept): leaf other | -4.28 | 1.01 | -4.25 | <0.01 |
| Aph-No | (Intercept): off plant | 0.01 | 0.17 | 0.08 | 0.93 |
| Aph-No | (Intercept): stem | -3.18 | 0.59 | -5.39 | <0.01 |
| Aph-No | (Intercept): stipule outside | -3.18 | 0.59 | -5.39 | <0.01 |
| Aph-No | Comparison: leaf bottom | 3.63 | 0.27 | 13.50 | <0.01 |
| Aph-No | Comparison: leaf other | 4.89 | 1.01 | 4.83 | <0.01 |
| Aph-No | Comparison: off plant | 0.77 | 0.19 | 4.11 | <0.01 |
| Aph-No | Comparison: stem | 4.37 | 0.60 | 7.35 | <0.01 |
| Aph-No | Comparison: stipule outside | 3.54 | 0.60 | 5.94 | <0.01 |
| Aph-Lys | (Intercept): leaf bottom | -1.33 | 0.26 | -5.17 | <0.01 |
| Aph-Lys | (Intercept): leaf other | -4.28 | 1.01 | -4.25 | <0.01 |
| Aph-Lys | (Intercept): off plant | 0.01 | 0.17 | 0.08 | 0.93 |
| Aph-Lys | (Intercept): stem | -3.18 | 0.59 | -5.39 | <0.01 |
| Aph-Lys | (Intercept): stipule outside | -3.18 | 0.59 | -5.39 | <0.01 |
| Aph-Lys | Comparison: leaf bottom | 2.89 | 0.36 | 8.11 | <0.01 |
| Aph-Lys | Comparison: leaf other | 2.38 | 1.18 | 2.01 | 0.04 |
| Aph-Lys | Comparison: off plant | -16.56 | 531.50 | -0.03 | 0.98 |
| Aph-Lys | Comparison: stem | 4.94 | 0.64 | 7.76 | <0.01 |
| Aph-Lys | Comparison: stipule outside | 3.18 | 0.67 | 4.75 | <0.01 |
| Lys-No | (Intercept): leaf bottom | 1.56 | 0.25 | 6.33 | <0.01 |
| Lys-No | (Intercept): leaf other | -1.90 | 0.62 | -3.06 | <0.01 |
| Lys-No | (Intercept): off plant | -15.55 | 322.37 | -0.05 | 0.96 |
| Lys-No | (Intercept): stem | 1.77 | 0.24 | 7.30 | <0.01 |
| Lys-No | (Intercept): stipule outside | <0.01 | 0.32 | <0.01 | 1.00 |
| Lys-No | Comparison: leaf bottom | 0.74 | 0.26 | 2.88 | <0.01 |
| Lys-No | Comparison: leaf other | 2.51 | 0.63 | 4.01 | <0.01 |
| Lys-No | Comparison: off plant | 16.33 | 322.37 | 0.05 | 0.96 |
| Lys-No | Comparison: stem | -0.57 | 0.26 | -2.23 | 0.03 |
| Lys-No | Comparison: stipule outside | 0.37 | 0.33 | 1.11 | 0.27 |

**Table S3: Summary of multinom model for aphid/ mummy positons, within treatments.** Values are coefficients +/- se. Comparison always to position stipule inside and with the number of aphids.

**A: Within *A. chaonia***

| **Level** | **Estimate** | **SE** | **z** | **p** |
| --- | --- | --- | --- | --- |
| (Intercept): leaf bottom | 0.94 | 0.12 | 7.59 | <0.01 |
| (Intercept): leaf other | -0.17 | 0.16 | -1.09 | 0.28 |
| (Intercept): off plant | -1.67 | 0.26 | -6.30 | <0.01 |
| (Intercept): stem | 0.78 | 0.13 | 6.11 | <0.01 |
| (Intercept): stipule outside | 0.40 | 0.14 | 2.92 | <0.01 |
| Number of mummies: leaf bottom | -2.27 | 0.29 | -7.95 | <0.01 |
| Number of mummies: leaf other | -4.11 | 1.02 | -4.03 | <0.01 |
| Number of mummies: off plant | 1.68 | 0.31 | 5.38 | <0.01 |
| Number of mummies: stem | -3.96 | 0.60 | -6.56 | <0.01 |
| Number of mummies: stipule outside | -3.58 | 0.60 | -5.91 | <0.01 |

**B: Within *L. fabarum***

| **Level** | **Estimate** | **SE** | **z** | **p** |
| --- | --- | --- | --- | --- |
| (Intercept):leaf bottom | 0.94 | 0.12 | 7.59 | <0.01 |
| (Intercept):leaf other | -0.17 | 0.16 | -1.09 | 0.28 |
| (Intercept):off plant | -1.67 | 0.26 | -6.30 | <0.01 |
| (Intercept):stem | 0.78 | 0.13 | 6.11 | <0.01 |
| (Intercept):stipule outside | 0.40 | 0.14 | 2.92 | <0.01 |
| Number of mummies:leaf bottom | -2.27 | 0.29 | -7.95 | <0.01 |
| Number of mummies:leaf other | -4.11 | 1.02 | -4.03 | <0.01 |
| Number of mummies:off plant | 1.68 | 0.31 | 5.38 | <0.01 |
| Number of mummies:stem | -3.96 | 0.60 | -6.56 | <0.01 |
| Number of mummies:stipule outside | -3.58 | 0.60 | -5.91 | <0.01 |

**Table S4: Summary of generalized linear mixed models to study the effect of treatment (*A. chaonia*, *L. fabarum* or no parasitoid exposure) on aphid/ mummy location at each position.** N=36 plants (12 per treatment).

Random effect of plant identity for each model for each position. Comparisons are with *A. chaonia* mummies. Treat: Tretament; Lfab: *L. fabarum*; noPa: No parasitoid

| **Postion** | **variance** | **sd** |
| --- | --- | --- |
| Leaf bottom | 0.77 | 0.88 |
| Leaf other | 0.29 | 0.54 |
| Off plant | 0.63 | 0.79 |
| Stem | 1.15 | 1.07 |
| Stipule inside | 0.62 | 0.79 |
| Stipule outside | 0.38 | 0.61 |

Fixed effects:

| **response** | **fixed effect** | **estimate** | **se** | **z** | **p** |
| --- | --- | --- | --- | --- | --- |
| Leaf bottom | (Intercept) | -2.38 | 0.39 | -6.09 | <0.001 |
| Leaf bottom | treat Lfab | 1.62 | 0.49 | 3.34 | <0.001 |
| Leaf bottom | treat noPa | 2.38 | 0.47 | 5.10 | <0.001 |
| Leaf other | (Intercept) | -5.27 | 1.02 | -5.17 | <0.001 |
| Leaf other | treat Lfab | 0.70 | 1.19 | 0.59 | 0.553 |
| Leaf other | treat noPa | 2.83 | 1.03 | 2.74 | 0.006 |
| Off plant | (Intercept) | -0.40 | 0.29 | -1.36 | 0.173 |
| Off plant | treat Lfab | -3.74 | 0.60 | -6.25 | <0.001 |
| Off plant | treat noPa | -1.84 | 0.38 | -4.92 | <0.001 |
| Stem | (Intercept) | -4.44 | 0.71 | -6.29 | <0.001 |
| Stem | treat Lfab | 4.32 | 0.79 | 5.51 | <0.001 |
| Stem | treat noPa | 2.55 | 0.77 | 3.31 | <0.001 |
| Stipule inside | (Intercept) | -0.36 | 0.29 | -1.25 | 0.213 |
| Stipule inside | treat Lfab | -2.30 | 0.45 | -5.11 | <0.001 |
| Stipule inside | treat noPa | -2.72 | 0.38 | -7.25 | <0.001 |
| Stipule outside | (Intercept) | -4.19 | 0.62 | -6.74 | <0.001 |
| Stipule outside | treat Lfab | 1.50 | 0.69 | 2.16 | 0.030 |
| Stipule outside | treat noPa | 1.59 | 0.65 | 2.45 | 0.014 |

**Table S5: Summary of generalized linear mixed models to analyse the effect of mummy position on hyperparasitism in the lab.** 81 observations (unique plant-position combinations) on 24 plants. Random effect of plant ID (variance ±sd): 0.44 ± 0.66. Comparison of fixed effects are with position leaf axil and species *A. chaonia*. Lfab: *L. fabarum.*

| **Fixed effect** | **Estimate** | **se** | **z** | **p** |
| --- | --- | --- | --- | --- |
| (Intercept) | -2.48 | 0.80 | -3.12 | 0.0018 |
| PositionLeaf bottom | 0.29 | 0.63 | 0.46 | 0.6434 |
| positionLeaf top | -0.68 | 0.59 | -1.15 | 0.2519 |
| positionStem | -0.88 | 0.68 | -1.30 | 0.1927 |
| speciesLfab | -1.12 | 0.83 | -1.35 | 0.1784 |
| Round | 2.10 | 0.51 | 4.09 | <0.0001 |
| PositionLeaf bottom:speciesLfab | 2.10 | 1.18 | 1.78 | 0.0755 |
| positionLeaf top:speciesLfab | 1.90 | 1.16 | 1.64 | 0.1011 |
| positionStem:speciesLfab | 2.66 | 1.16 | 2.29 | 0.0222 |

**Table S6: Summary of generalized linear mixed models to analyse the effect of mummy position in the field.** Comparison of fixed effects are with leaf axil.

**A: Proportion of parasitoids out of all hatched wasps**, 41 observations (unique plant-position combinations) on 12 plants in 3 pots.

Random effects:

| **Random effect** | **Variance** | **sd** |
| --- | --- | --- |
| Plant ID:Pot ID | 0.48 | 0.70 |
| Pot ID | 1.17 | 1.08 |

Fixed effects:

| **Fixed effect** | **Estimate** | **se** | **z** | **p** |
| --- | --- | --- | --- | --- |
| (Intercept) | -0.43 | 0.79 | -0.54 | 0.5885 |
| positionLeaf bottom | 0.42 | 0.62 | 0.67 | 0.5040 |
| positionLeaf top | 0.78 | 0.61 | 1.27 | 0.2043 |
| positionStem | 0.32 | 0.88 | 0.36 | 0.7167 |

**B: Proportion of mummies retrieved out of all glued mummies**, 48 observations (unique plant-position combinations) of 12 plants in 3 pots.

Random effects:

| **Random effect** | **Variance** | **sd** |
| --- | --- | --- |
| Plant_ID:Pot_ID | 0.29 | 0.54 |
| Pot_ID | <0.01 | 0.05 |

Fixed effects:

| **Fixed effect** | **Estimate** | **se** | **z** | **p** |
| --- | --- | --- | --- | --- |
| (Intercept) | 0.55 | 0.30 | 1.83 | 0.0670 |
| PositionLeaf bottom | 0.47 | 0.37 | 1.28 | 0.2004 |
| positionLeaf top | 0.33 | 0.36 | 0.91 | 0.3643 |
| positionStem | -1.29 | 0.36 | -3.55 | 0.0004 |

**C: Overall parasitoid survival (proportion of hatched parasitoids out of all glued mummies)**, 48 observations (unique plant-position combinations) of 12 plants in 3 pots.

Random effects:

| **Random effect** | **Variance** | **sd** |
| --- | --- | --- |
| plant_ID:pot_ID | 0.0734 | 0.2709 |
| pot_ID | 0.5714 | 0.7559 |

Fixed effects:

| **Fixed effect** | **Estimate** | **se** | **z** | **p** |
| --- | --- | --- | --- | --- |
| (Intercept) | -1.5100 | 0.5400 | -2.7800 | 0.0054 |
| positionLeaf bottom | -0.0900 | 0.4300 | -0.2200 | 0.8277 |
| positionLeaf top | 0.1700 | 0.4200 | 0.4200 | 0.6739 |
| positionStem | -1.3300 | 0.5500 | -2.3900 | 0.0168 |
