# Supplementary material for: A Parasitoid of Aphids Manipulates Host Mummification Site, With Effects on Survival but Not Hyperparasitism
Source: Ecol Evol. 2026 Feb 11;16(2):e72764. doi: 10.1002/ece3.72764 (PMC12894770; doi:10.1002/ece3.72764)
Supplement: Supplementary file 2 — Figure S1: ece372764‐sup‐0002‐FigureS1.pdf. [file ECE3-16-e72764-s002.docx]

**Figure S1: Proportion of aphids or mummies found at different positions of the plant within each treatment.** Error bars represent 95% CI.
